# Arrhythmogenic Right Ventricular Cardiomyopathy/Dysplasia: An Updated Review of Diagnosis and Management

**DOI:** 10.7759/cureus.5381

**Published:** 2019-08-13

**Authors:** Yasar Sattar, Hafez Mohammad Abdullah, Elham Neisani Samani, Madhura Myla, Waqas Ullah

**Affiliations:** 1 Internal Medicine, Icahn School of Medicine at Mount Sinai, New York, USA; 2 Internal Medicine, University of South Dakota Sanford School of Medicine, Sioux Falls, USA; 3 Internal Medicine, Yale University, New Haven, USA; 4 Cardiology, University of New Mexico, New Mexico, USA; 5 Internal Medicine, Abington Hospital-Jefferson Health, Abington, USA

**Keywords:** arrhythmogenic right ventricular cardiomyopathy, arrhythmogenic right ventricular dysplasia, pathophysiology, diagnosis, treatment

## Abstract

Arrhythmogenic right ventricular cardiomyopathy/dysplasia (ARVC/D) is a condition caused by the replacement of the normal right ventricular myocardium with fibrofatty tissue. ARVC/D can present with a variety of clinical conditions including right ventricular dysfunction, ventricular tachyarrhythmias, sudden cardiac arrest, and sudden cardiac death (SCD). Since the first report of ARVC/D in 1982, many advances have been made in the diagnosis, genetic findings for pathology, and treatment. The 2010 International Task Force diagnostic criteria distinguish between major and minor criteria and focus on gross structural changes, microscopic changes, repolarization defects, conduction defects, arrhythmias, and family history. Implantable cardiac defibrillators and catheter ablation of the endocardium and epicardium with electromagnetic mapping have emerged as successful tools in the treatment and prevention of ventricular tachyarrhythmias and SCD. This review discusses the pathophysiology, genetics, diagnosis, and treatment advances in ARVC/D.

## Introduction and background

Arrhythmogenic right ventricular cardiomyopathy/dysplasia (ARVC/D) is an autosomal dominant condition and was first reported in 1982. ARVC/D is defined as fibrofatty infiltration in the cardiac myocytes of the right-most ventricle with a predisposition to anatomical, contractility, and electrical rhythm abnormalities with life-threatening complications [1]. The fibrofatty replacement is not just limited to the right ventricle (RV); in rare instances, severe cases can have fibrofatty involvement of the left ventricle (LV) [1]. The prevalence of ARVC is 1:2500 or 1:5000, considering sudden death (which remains unrecognized) [2]. ARVC is three times more prevalent in men than women [3]. Causative mutations in a variety of desmosomes genes have been identified, including desmoglein, desmoplakin (DSP), plakoglobin, plakophilin-2 (PKP-2), and desmocollin [4]. Clinical manifestations can be broadly divided into contractility and electrical rhythm abnormalities. In an early phase, individuals are often asymptomatic. Anatomical abnormalities may be absent or localized with or without arrhythmia. As the anatomical changes progress, symptomatic cardiac dysfunction can be revealed by imaging and rhythm abnormalities. The diagnosis of ARVC/D remains a clinical challenge. There is no precise clinical or paraclinical test to verify or exclude ARVC/D. The 2010 International Task Force criteria offer guidance on the diagnosis of ARVC/D. In this comprehensive review, we summarize the progress that has been made in understanding the pathogenesis and the diagnostic and therapeutic modalities of ARVC/D.

## Review

Clinical presentation

ARVC/D is inherited as an autosomal dominant disease and constitutes variable expressivity and incomplete penetrance [4]. Many patients with ARVC/D, particularly those with sporadic cases, remain clinically asymptomatic for decades, making the condition difficult to diagnose [2]. When the condition is symptomatic, there are a variety of phenotypic expressions of ARVC/D [2]. The disease presents symptomatically in the second to fourth decades of life. ARVC/D can present as any symptom of ventricular tachyarrhythmias, including palpitations, dizziness, syncope, and sudden cardiac death (SCD). The first and final presentation can be SCD [3]. The span of ventricular arrhythmias includes premature ventricular complexes, ventricular tachycardia (VT), and ventricular fibrillation [2]. ARVC/D is divided into four stages based on long-term follow-up. The first stage is “subclinical” in which we have concealed functional and structural changes, even though SCD can be the first manifestation in this stage. The second stage is “overt electrical” with electrocardiogram (EKG) findings of RV arrhythmias and both structural and functional abnormalities. The third stage is “right-ventricle dysfunction” with the presence of severe RV involvement and the absence of LV involvement. The fourth stage is “biventricular or late” with severe involvement of both the RV and LV [5]. ARVC/D is well recognized to be linked with exercise and can present early, with worse symptoms in patients playing competitive sports and having underlying desmosomal mutations [2]. In a study by Ruwald et al., competitive sports double the risk of ventricular tachyarrhythmias and SCD compared with recreational sports [6]. ARVC has been found to account for sudden unexplained death in 11-22% of young athletes and 10% of young people [7].

Genetics

ARVC/D has a mutation in the intercellular junction and adhesive proteins like desmosome. The genetic mutation involving desmosomes are present in almost 60% of cases [8]. Forty percent of cases can be genotype negative; in fact, negative genotype cases can have cadherin-2 protein mutations in generations [9]. The ARVC/D mutation was first described to have plakoglobin autosomal recessive mutation as part of Naxos disease that affects the heart, nail tissue, and skin [10]. DSP was the first gene found to be linked with the autosomal dominant form of ARVC/D, and recessive mutations of DSP are found in a cardiocutaneous disease called Carvajal syndrome [11]. At the intercalate disc junctions, a protein known as PKP2 mediates the plakoglobin and DSP. The PKP2 protein encoded by the PKP2 gene is the most common autosomal dominant mutations found in ARVC/D, and its prevalence can range from 7% to 51% [12]. The three significant mutations constituting 90% of the mutations in ARVC include the PKP2, DSP, and desmoglein-2 (DSG2) genes [13]. Mutant DSG2 is unable to initiate desmosome assembly, preceding the robust fibrofatty change in the RV, leading to early-onset and severe ARVC/D [14]. Patients with a genotype negative for desmosomes frequently have mutations in cadherins. In a study by Mayosi et al., one of the variants of CDH2 (c.1219G>A, p.Asp407Asn) was found in 73 cases of negative genotype cases of ARVC/D and may have a role in its pathogenesis [9]. Other extra-desmosomal genes involved in ARVC/D include transforming growth factor β3, cardiac ryanodine-2 receptor, and the transmembrane protein 43 genes [15-17].

Pathophysiology

The hallmark feature of ARVC/D is right ventricular fibrofatty replacement that impedes electrical conduction, leading to tachyarrhythmias [2]. ARVC/D pathophysiology encircles the genetically abnormal desmosomal protein, causing myocyte detachment and death. The desmosome complex consists of three main components: DSP (which holds the intermediate filaments), the transmembrane proteins (cadherins; including desmocollin-2 and DSG2), and linkage proteins (e.g., PKP-2 and plakoglobin, which connects DSP and cadherin tails) [18]. As a result of mutations in desmosome proteins like DSP, the plakoglobin is set free, fails to integrate into desmosome complex, and shifts from the intercellular junction to the cytosol and nucleus. Increased plakoglobin concentration in the nucleus and cytosol causes downregulation of canonical Wnt/β‐catenin pathway by inhibiting the formation of a complex of β‐catenin and T-cell factor-lymphocyte-enhancing factor. This downregulation of the complex formation leads to enhanced adipogenesis, fibrogenesis, and myocyte apoptosis [19]. As a result of the altered Wnt/β‐catenin pathway, the increase in the expression of fibrogenic genes and the stimulation of the fibroblast growth factors result in increased fibrogenesis [20]. The fibrotic conversion of the heart initially starts in the epicardium of the RV and migrates towards the endocardium and RV free wall, resulting in the thinning of trabeculae and aneurysmal dilatation migrating from the subtricuspid region to the infundibular region and apex of the RV [18-20].

**Figure 1:**
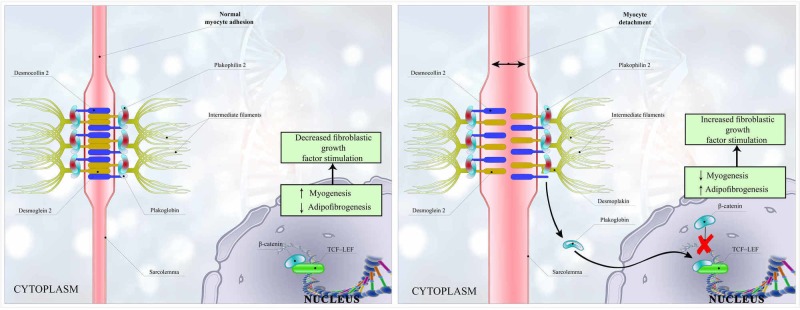
Intercellular junctions and intracellular growth signaling in arrhythmogenic right ventricular cardiomyopathy/dysplasia A: Normal attachment of desmosomes parts including the connection of desmocollin and desmoglein, plakoglobin, and plakophilin-2 connected to each other and intermediate filaments. β‐catenin inhibits fibrogenesis by inhibiting TCF-LEF. B: Separation of desmocollin and desmoglein causing myocyte detachment, which dislodges the plakoglobin. Plakoglobin causes separation of β‐catenin and TCF-LEF, which, in turn, stimulates fibrogenesis and decreases myogenesis. Abbreviation: TCF-LEF, T-cell factor–lymphocyte-enhancing factor.

Furthermore, the uncoupling of the myocyte and detachment in ARVC/D is further enhanced by factors increasing afterload and wall stress, including exercise as a significant risk factor. Furthermore, inflammatory or infectious theories have also been proposed as precipitating or facilitating gene expression. Campian et al. found that patients with ARVC/D had higher plasma levels than controls of the proinflammatory cytokines interleukin (IL)-1β, IL-6, and tumor necrosis factor-α [21]. Another theory supporting the role of inflammation caused by an infection in ARVC revealed that Coxsackievirus B3 develops myocardial cell necrosis and subsequent ARVC/D [22]. Recently, Lombardi et al. found the disrupted differentiation of cardiac stem cells as a potential mechanism of ARVC/D [23]. A simplified version of the mechanism of pathogenesis in ARVC/D is shown in Figure 1.

Diagnosis

Clinical diagnosis of ARVC/D remains challenging due to nonspecific manifestations and nondefinitive diagnostic tests. The histopathology findings of transmural fibrofatty replacement of RV myocardium at necropsy are necessary to confirm a diagnosis of ARVC/D. However, because of the limitations of diagnosis in 1994, the first criteria to diagnose ARVC was introduced as the International Task Force Criteria based on family history, biopsy results, anatomical abnormalities, and EKG abnormalities. Unfortunately, the initial diagnostic criteria lacked sensitivity to detect early familial cases [20-22]. Therefore, the sensitivity of the diagnostic criteria improved with a revision in 2010. These criteria updated in the 2010 International Task Force Criteria are divided into major and minor categories. The “definitive” diagnosis of ARVC is made by the inclusion of two major criteria, one major and two minor criteria, or four minor criteria. The “borderline” diagnosis includes one major and one minor criterion, or three minor criteria from different categories. “Possible” ARVC includes one major criterion or two minor criteria [24]. Following is a detailed discussion of the 2010 International Task Force Criteria.

Electrocardiogram

**Figure 2:**
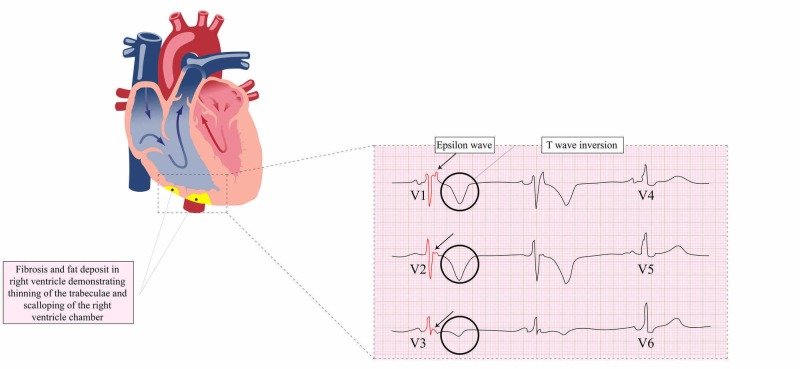
Fat deposits in the right ventricle and EKG changes in ARVC, including Epsilon waves and T-wave inversions in leads V1-V3 Abbreviations: EKG, electrocardiogram; ARVC, arrhythmogenic right ventricular cardiomyopathy.

Fibrofatty infiltration of the myocardium creates repolarization and conduction defects that can present as a potentially life-threatening VT on EKG. The alterations on EKG vary according to the extent and localization of the disease. In the right precordial leads (V1-V6), T-wave inversions are present in 87% of patients with ARVC, which are directly related to RV dilatation and may extend to the left precordium with time-related effects on the left-sided extension [7]. According to the 2010 Revised Task Force guidance, the major EKG criteria for patients older than 14 years include inverted T-waves in precordial leads V1-V3 without the right bundle branch block (RBBB)/QRS > 120 msec and Epsilon waves in precordial leads V1-V3. The minor EKG criteria in patients older than age 14 include inverted T-waves in leads V1-V2, or V4, V5, V6 in the absence of complete RBBB (CRBBB), or inverted T-waves in leads V1-V4 in the presence of CRBBB. Other minor criteria of diagnosis include late potentials by averaged signal EKG in at least one of the following three parameters in the absence of QRS prolongation > 110 msec: (1) filtered QRS duration ≥ 114 msec, (2) Duration of terminal QRS < 40 μV as ≥ 38 msec, and (3) RMS voltage of terminal 40 msec as ≥ 20 μV. Other minor criteria include QRS ≥ 55 msec from the nadir of the S-wave to the point where the QRS wave ends, including R', in V1-V3, in the absence of CRBBB, nonsustained/sustained VT of the right ventricular site with left bundle branch pattern with inferior axis deviation or unknown axis, and ventricular ectopic systolic beats > 500 in 24 hours recorded on a Holter monitor [24]. A study by Jain et al. revealed that T-wave inversions through V3 with the absence of CRBBB or the presence of an incomplete RBBB pattern is the single best optimally sensitive and specific finding on EKG [25]. This study also revealed that in patients with CRBBB, an R/S ratio of <1 in V1 is the optimally sensitive and specific EKG feature [25]. Several nonspeciﬁc ST abnormalities can be found from ﬂat precordial T-waves to Brugada-like EKGs. J-point elevation > 1 mm in at least two inferior and lateral leads is a common ﬁnding in >20% of EKGs, reﬂecting slow depolarization [26]. These criteria are highly speciﬁc but lack sensitivity, especially in early stages when RV dilatation/dysfunction may be absent. A retrosternal position of RV and its geometry make the evaluation difficult. Also, the assessment of wall motion abnormalities requires speciﬁc expertise [27]. The EKG spectrum of specific findings seen in ARVC/D is shown in Figure 2.

Echocardiography

Two-dimensional (2D) echocardiography is commonly used for the diagnosis and follow-up evaluation of ARVC. Dilatation and reduced regional or global RV function are characteristic findings. According to the 2010 Revised Task Force guidance, the major and minor criteria for the diagnosis of ARVC based on echocardiography consist of RV dyskinesia/akinesia or aneurysm in addition to a right ventricular outflow tract diameter of ≥32 mm (major)/≥ 29 to < 32 mm (minor) in the parasternal long axis (PLAX), ≥ 36 mm (major)/≥ 32 to 36 mm (minor) in the parasternal short axis (PSAX), or axis correction for body surface area (BSA) including PLAX/BSA ≥ 19 mm/m2 (major)/≥ 16 to <19 mm/m2 (minor) or PSAX/BSA ≥ 21 mm/m2 (major)/≥ 18 to < 21 mm/m2 (minor) or fractional area change of ≤ 33% (major)/> 33% to ≤ 40% mm/m2 (minor) [24]. Furthermore, Peter et al. hypothesize that intracardiac echocardiography in ARVD provides better additional information about tissue characterization and can be an add-on with angiography [28].

Cardiac multidetector computed tomography

Cardiac multidetector computed tomography (MDCT) can influentially detect most of the qualitative and quantitative findings of the RV in patients with ARVD/C. MDCT diagnostic findings for ARVC/D in RV includes increased trabeculation, intramyocardial fat, and scalloping [29]. Limitations of MDCT include motion artifacts, contrast-mediated reaction, and radiation exposure [29].

Cardiac magnetic resonance imaging

Cardiac magnetic resonance imaging (MRI) is a potential gold standard for distinguishing muscle from fat/fibrosis besides assessing ventricular volumes and regional function [24]. Visualizing both structural and functional cardiac abnormalities with the use of late gadolinium enhancement makes MRI the preferred imaging technique [30]. According to the 2010 Revised Task Force, the major and minor criteria for the diagnosis of ARVC based on MRI findings include RV akinesia/dyskinesia or dyssynchronous RV contraction in addition to RV end-diastolic volume of ≥ 110 mL/m2 in male patients and ≥100 mL/m2 in female patients (major)/≥ 100 mL/m2 to <110 mL/m2 in male patients and ≥ 90 to <100 mL/m2 in female patients (minor criteria) and RV ejection fraction of ≤ 40% (major)/>40% to ≤ 45% (minor) [24]. Unfortunately, cardiac MRI is not a good option for patients on implanted cardiac devices, but MDCT is an acceptable alternative [30].

Cardiac angiography

Angiography allows for an accurate assessment of wall motion, RV volume, and RV ejection fraction. It provides other findings relevant to ARVC/D, including global and regional dilatation, aneurysms, contrast evacuation, and trabecular size and structure. RV wall motion was significantly reduced at the apex of the RV and to some extent at the tricuspid and inferior wall regions [31]. Patients with ARVC/D can also have reduced hyperemia myocardial blood flow and increased vascular resistance in the coronary arteries [31-32]. RV akinesia, dyskinesia, and aneurysm on RV angiography are major criteria in the 2010 Revised Task Force diagnostic criteria [24].

Three-dimensional electroanatomical voltage

Three-dimensional (3D) voltage mapping assists in diagnosing ARVC/D by showing low-voltage areas that were linked with the fibrofatty myocardial replacement of the RV and assists in differentiating among patients with inflammatory cardiomyopathy who fulfill Task Force diagnostic criteria but depict a preserved electrogram voltage, making ARVC less likely [33]. Three-dimensional voltage mapping increases the diagnostic yield of endomyocardial biopsy from the focal areas of low voltage [34].

Nuclear imaging

Programmed cell death in the RV is considered one of the main features of ARVC/D. Campian et al. revealed that ARVC/D has chamber-specific apoptosis detectable in vivo with Tc-annexin V scintigraphy (99m). Nuclear imaging study aids in diagnosis, allows disease progression monitoring, and helps in checking responses to a variety of treatments [35].

Endomyocardial biopsy

A biopsy is invasive but holds a pivotal role in the diagnosis of ARVC/D. The safer route of endomyocardial biopsy is 2D echocardiography-guided transfemoral right ventricular biopsy [36]. Per the 2010 Revised Task Force Criteria for diagnosis, at least one sample with fibrous replacement of the RV free wall with or without fatty replacement in addition to <60% residual myocytes is a major criterion, whereas 60-75% residual myocytes is a minor criterion [24].

Family history

A family history of ARVC/D is a risk factor. According to the 2010 Revised Task Force Criteria for diagnosis, the major criteria include ARVC/D in a first-degree relative, confirmed either by the current task force criteria or pathologically, and identification of a pathogenic mutation in the individual. The minor criteria include sudden premature death (<35 years) due to suspected ARVC/D or a history of ARVC/D which cannot be practically determined in a first-degree relative or ARVC/D in a second-degree relative confirmed by the current task force criteria or pathologically [24].

Inflammatory and cardiac biomarkers

Biomarkers for heart dysfunction like brain natriuretic peptide (BNP) is increased in ARVC/D. BNP levels correlate with the severity of both the RV dysfunction and area containing electrograms with delayed components (e.g., the arrhythmogenic substrate area). In ARVC/D patients, RV wall inflammation can be detected noninvasively by a combination of plasma levels of inflammatory cytokines and cardiac (67) Ga scintigraphy [21].

Treatment

A key goal of therapy is to prevent life-threatening arrhythmia and improve the quality of life by reducing heart failure symptoms. Therapeutic options consist of lifestyle modifications, pharmacological treatment, radiofrequency ablation (RFA), implantable cardioverter-defibrillator (ICD), and transplantation.

Lifestyle modifications

Notably, the essential recommendation for patients with ARVD is lifestyle modification, particularly exercise restriction. Endurance exercise aggravates the progression of the disease in both humans and animal models [37]. Physical exercise increases wall stress to a greater extent in the ventricles. As competitive sports activity increases the risk of SCD by fivefold in adolescents and young adults with ARVC, early identification of affected athletes by preparticipation screening from the age of 11 to 12 years may be life-saving [38].

Pharmacological therapy

Antiarrhythmic drugs (primarily beta-blockers) and amiodarone have been used for symptomatic patients who are not candidates for ICD or as an adjunct therapy. Sotalol was the most effective drug, with an overall success rate of 68%. Amiodarone, alone or in combination with beta-blockers, has been shown as an alternative approach [39]. In patients with RV or biventricular heart failure, treatment consists of diuretics, angiotensin-converting enzyme inhibitors/angiotensin receptor blockers, digitalis, and anticoagulants [40]. In a double-blinded clinical trial of a blockade of the renin-angiotensin-aldosterone system in patients with ARVD conducted by Morel et al., 120 patients will be followed every six months over three years and will get either ramipril or placebo. This study will look at a decrease in right and left ventricular deterioration and arrhythmia burden in patients with ARVD treated with Ramipril or placebo [41].

Radiofrequency ablation

Radiofrequency catheter ablation has a crucial role in improving the quality of life by reducing symptoms and VT recurrence. Patients with ARVC/D and VT after endocardial ablation have a more extensive epicardial area of electrogram abnormalities and frequently have basal right ventricular wall thickening [42]. Epicardial substrate and 3D electroanatomical VT mapping identify epicardial targets, and ablation of epicardial focus results in better VT control [43]. In a clinical trial by Garcia et al., 13 patients underwent endocardial-epicardial sinus voltage mapping and epicardial VT focus ablation after failing endocardial VT ablation [44]. In this study, 10 of 13 patients had no VT events, and 2 patients had only a single VT event at 2 months and 38 months follow-up, respectively [44]. ARVC/D with VT after endocardial ablation has VT originating from the epicardial area, which should be mapped and ablated for better outcomes.

Furthermore, in a study by Phillips et al., 87 patients with ARVC/D underwent 175 RFAs over 19 years with an inception date of 1992 at 80 different electrophysiological centers [42]. They reported that after a mean follow-up of 88.3 ± 66 months, the overall VT-free percentage was 47%, 21%, and 15%, at 1, 5, and 10 years, retrospectively, and VT-free cumulative percentage following epicardial RFA was 64% and 45% at one and five years, respectively [42]. However, RFA must be conducted by an expert, as the risks of significant complications related to epicardial ablation are considerable [42].

Cardioverter-defibrillator implantation

ICDs remain the sole proven therapy for preventing SCD in patients with ARVC [43]. Patients with unexplained syncope, family history of sudden death, and extensive disease are potential candidates for ICD use. ICD therapy is a practical approach for primary and secondary prevention of SCD in patients with ARVD/C [43]. However, ICD therapy is not without risk in patients with ARVD/C [45-46]. The mortality rate in patients with ARVD/C after ICD implantation is low. Appropriate ICD interventions occur at a rate of 9.5% per year [47]. In a study by Corrado et al., 132 patients with ARVC had ICD and were followed up at 36 months; the actual survival rate was 76% [47]. Wietholt et al. reported that an antitachycardia pacing technique by ICD showed favorable outcomes and terminated up to 91% of the hemodynamically stable VTs in patients [48].

Heart transplantation

Finally, heart transplantation is the last therapeutic option in ARVC patients who have a refractory arrhythmia or heart failure [49].

## Conclusions

Our knowledge of ARVC has expanded. While the pathogenesis of ARVC is now well understood, the early diagnosis of arrhythmias can be made by a constellation of genetic, imaging, and biopsy results. Among the available treatments is a new practice of catheter ablation with a focus of ablating both endocardium and epicardium foci, which helps prevent tachyarrhythmias. Modern technologies like 3D electroanatomical VT mapping highlight the areas in need of ablation. Future studies that explore noninvasive, molecular, and genetic treatments are needed.
